# Supplementary figures and images for: A microRNA that controls the emergence of embryonic movement
Source: eLife. 2024 Jun 13;13:RP95209. doi: 10.7554/eLife.95209 (PMC11175612; doi:10.7554/eLife.95209)

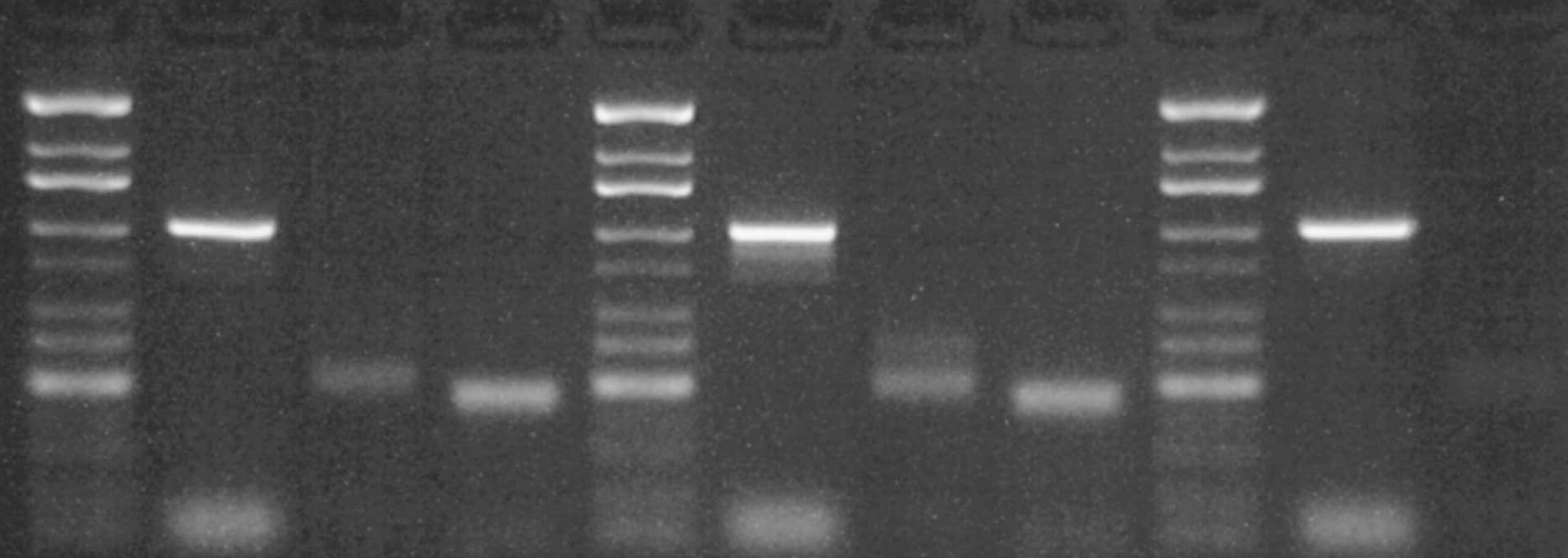

Supplement: Figure 3—source data 1. — 3 p transcript is non-specific due to amplification of other identical miR-2 family 3 p transcripts. [file elife-95209-fig3-data1.pdf]

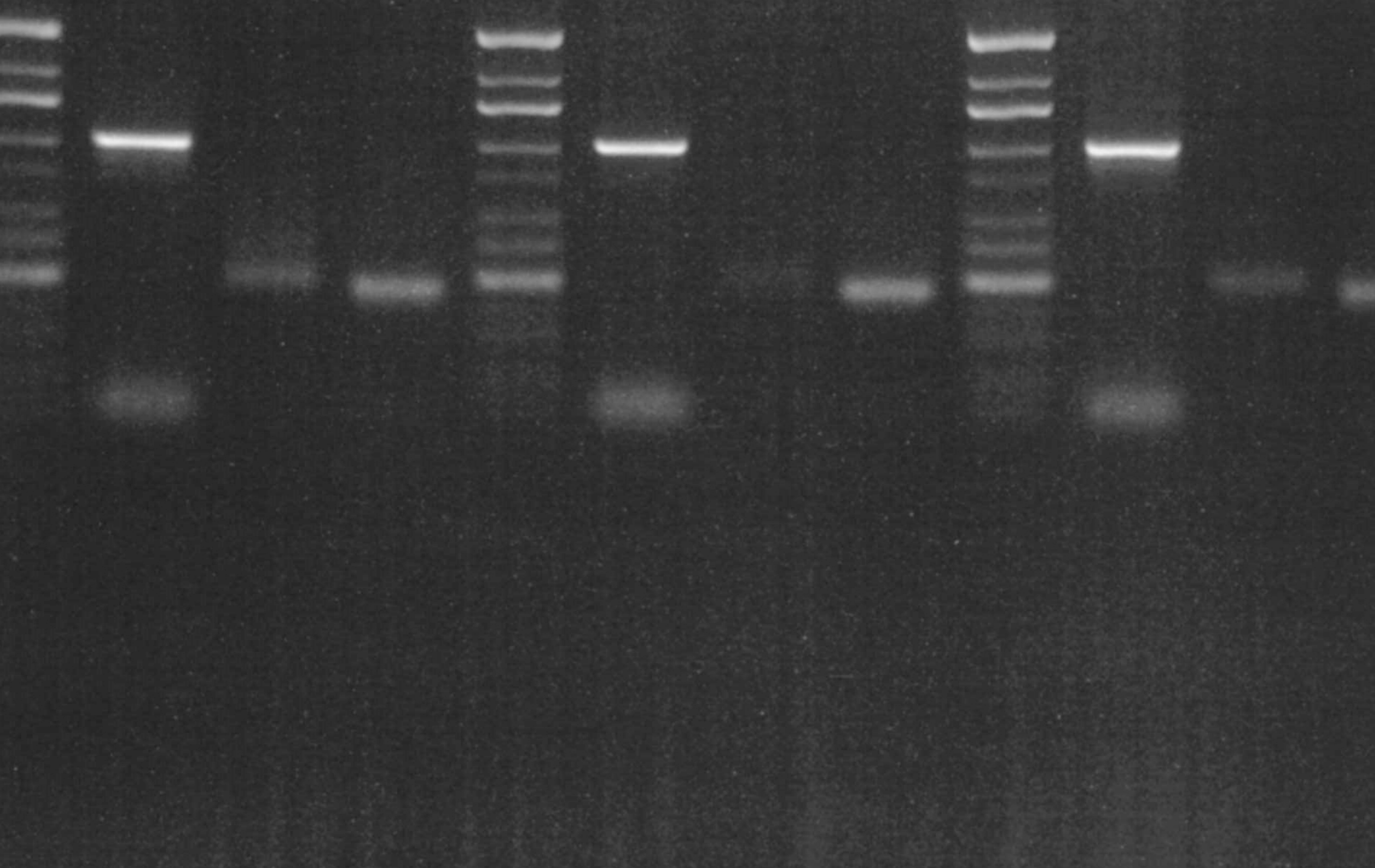

Supplement: Figure 4—source data 4. — 3 p transcript is non-specific due to amplification of other identical miR-2 family 3 p transcripts. [file elife-95209-fig4-data4.pdf]
